# A feature selection method based on the Golden Jackal-Grey Wolf Hybrid Optimization Algorithm

**DOI:** 10.1371/journal.pone.0295579

**Published:** 2024-01-02

**Authors:** Guangwei Liu, Zhiqing Guo, Wei Liu, Feng Jiang, Ensan Fu

**Affiliations:** 1 College of Mining, Liaoning Technical University, Fuxin, Liaoning, China; 2 College of Science, Liaoning Technical University, Fuxin, Liaoning, China; University 20 Aout 1955 skikda, Algeria, ALGERIA

## Abstract

This paper proposes a feature selection method based on a hybrid optimization algorithm that combines the Golden Jackal Optimization (GJO) and Grey Wolf Optimizer (GWO). The primary objective of this method is to create an effective data dimensionality reduction technique for eliminating redundant, irrelevant, and noisy features within high-dimensional datasets. Drawing inspiration from the Chinese idiom “Chai Lang Hu Bao,” hybrid algorithm mechanisms, and cooperative behaviors observed in natural animal populations, we amalgamate the GWO algorithm, the Lagrange interpolation method, and the GJO algorithm to propose the multi-strategy fusion GJO-GWO algorithm. In Case 1, the GJO-GWO algorithm addressed eight complex benchmark functions. In Case 2, GJO-GWO was utilized to tackle ten feature selection problems. Experimental results consistently demonstrate that under identical experimental conditions, whether solving complex benchmark functions or addressing feature selection problems, GJO-GWO exhibits smaller means, lower standard deviations, higher classification accuracy, and reduced execution times. These findings affirm the superior optimization performance, classification accuracy, and stability of the GJO-GWO algorithm.

## 1. Introduction

With the continuous evolution and innovation in computer science technology and storage hardware and their widespread applications in fields such as finance, social media, and biomedicine, various forms of unstructured data have experienced exponential growth [1]. However, these unstructured data types typically contain numerous redundant, irrelevant, and noisy features, making subsequent data mining and scientific research processes challenging [2]. Therefore, the rational and practical identification of optimal feature subsets within these unstructured data collections is essential for subsequent data engineering research.

Feature selection (FS) is a method employed to reduce the dimensionality of data by eliminating a substantial number of redundant, irrelevant, and noisy features from the original dataset while endeavoring to retain all the essential attributes [3–5]. Based on the variations in search and evaluation techniques, feature selection is traditionally classified into three fundamental categories: filter, wrapper, and embedded methods [6, 7]. Notably, a trade-off characterizes the relationship between filter and wrapper methods. While filter methods are computationally more efficient, wrapper methods excel in feature selection tasks by incorporating the classification model’s feedback [8]. Consequently, this paper’s primary research focuses on the wrapper-type feature subset search process.

The crux of addressing the feature selection problem lies in searching for and evaluating feature subsets [9]. This search for a feature subset can be seen as a combinatorial optimization problem, traditionally approached through exhaustive techniques or heuristic approaches. However, the perpetual accumulation of data has sparked the predicament known as the “curse of dimensionality” [10], rendering traditional methods that rely on exhaustive sampling of every data point or heuristic techniques impractical [11]. In essence, selecting informative feature subsets from high-dimensional data presents notable challenges, necessitating the development of effective feature selection methods to efficiently reduce the original data into a lower-dimensional space [12, 13].

Recently, metaheuristic algorithms have emerged as a preferred tool for tackling combinatorial optimization problems [8, 14–16]. These algorithms are highly regarded for their straightforward heuristics, robust global search capabilities, and insensitivity to parameter settings, making them versatile solutions across various domains [17–19]. When employed as search strategies for feature subset selection to address feature selection challenges, metaheuristic algorithms prove advantageous in circumventing the issues associated with traditional optimization methods.

Consequently, various metaheuristic algorithms have been applied to feature selection problems, yielding meaningful research outcomes [20–26]. Notable examples of these algorithms include Genetic Algorithm [27–29], Particle Swarm Optimization (PSO) [30–32], Ant Colony Optimization (ACO) [33–36], Artificial Bee Colony Algorithm (ABC) [37–39], Grey Wolf Optim1izer (GWO) [40–46], Whale Optimization Algorithm (WOA) [47–53], Multi-verse Optimizer (MVO) [41, 54–56], Salp Swarm Algorithm (SSA) [57–62], Atom Search Optimization (ASO) [63, 64], Harris Hawks Optimizer (HHO) [65–68], Grasshopper Optimization Algorithm (GOA) [69–71], and Sooty Tern Optimization Algorithm (STOA) [72, 73], among others. Table 1 summarizes select metaheuristic algorithms employed for feature selection in the past three years.

**Table 1:** Typical metaheuristic algorithms used for feature selection in the past three years.

Algorithm	Technique	Methodology	Result
GA [29]	Genetic Algorithm	GA+KNN	GA+KNN approach on a lung cancer database reveals 100% accuracy.
PSO [74]	Particle Swarm Optimization	SS-PSO	SS-PSO is a highly competitive method for high-dimensional FS.
ACO [36]	Ant Colony Optimization	AMFSA	AMFSA is effective in achieving an excellent feature subset with great classification efficiency by 15 multilabel datasets.
GWO [45]	Grey Wolf Optimization	ABGWO	ABGWO has the most advantages in classification accuracy, feature subset size and calculation time.
WOA [49]	Whale Optimization Algorithm	IWOAIKFS	IWOAIKFS has better classification and robustness.
ABC [37]	Artificial Bee Colony Algorithm	SEABC	SEABC algorithm achieves better classification results.
ASO [75]	Atom Search Optimization	CEOAS	CEOAS algorithm achieved recognition accuracy rates of 98.01%, 98.72%, 84.62% and 74.25% respectively on SAVEE, EmoDB, RAVDESS and IEMOCAP.
GSA [76]	Gravitational Search Algorithm	GPSOGSA	GPSOGSA could escape from local optimum and converges faster than the PSO, GSA and PSOGSA algorithms.
MVO [77]	Multi-verse Optimization	MVO+RF	MVO+RF approach gives high accuracy compared to existing techniques like PSO and BAT
SSA [62]	Salp Swarm Algorithm	TLBO-SSA	TLBO-SSA archived an accuracy of 98.46% with 98.81% sensitivity, 98.08% specificity, 0.9852 F-score, 0.9692 Cohen’s kappa coefficient, and area under curve AZ = 0.997 ± 0.001.
ALO [78]	Ant Lion Optimizer	HBALO	HBALO achieved good performance on 10 UCI benchmark datasets and COVID-19 patient health datasets.
BOA [79]	Butterfly Optimization Algorithm	PIL-BOA	PIL-BOA is superior to other competitors in term of classification accuracy.
HHO [65]	Harris Hawks Optimizer	LIL-HHO	LIL-HHO shows superior performances on most cases relative to the basic HHO and other compared meta-heuristic algorithms.
SCA [80]	Sine Cosine Algorithm	ImpSCA	Three versions of ImpSCAs (except ImpSCA 3) outperform the original SCA in 80% of the datasets.
GOA [70]	Grasshopper Optimization Algorithm	LAGOA	LAGOA has been used in standard disease datasets of the UCI machine learning repository and the results prove its superiority over other state-of-the-art methods on these datasets.
SMA [81]	Slime Mould Algorithm	GBOSMA	GBOSMA has better optimization performance, search speed and stability when solving global optimization and feature selection problems.
STOA [73]	Sooty Tern Optimization Algorithm	mSTOA	mSTOA has the ability to extract the best feature subset.
…	…	…	…

The metaheuristic algorithms mentioned above have each made valuable contributions to feature selection at different points in time, capitalizing on their strengths. Nevertheless, as per the No-Free Lunch theorem [82], it is essential to acknowledge that no single algorithm can universally address all optimization problems. This recognition drives researchers towards a continuous quest for more advanced and versatile algorithms capable of addressing diverse challenges.

The Golden Jackal Optimization (GJO) algorithm [83] has emerged as a promising contender among these metaheuristic algorithms. GJO draws inspiration from the hunting behavior of golden jackals and is renowned for its minimal parameterization, swift search capabilities, and remarkable global exploration potential. It has found applications across a spectrum of complex problem domains. However, the foundational GJO algorithm grapples with certain limitations when confronted with intricate optimization challenges, particularly issues associated with local optima and diminished solution precision. Therefore, a significant impetus behind this study is enhancing the GJO algorithm to improve its optimization performance. Furthermore, another crucial motivation is to explore the application of this enhanced GJO algorithm in tackling feature selection problems. To be specific, the main contributions of this paper are outlined as follows:

1. Drawing inspiration from the Chinese idiom “Chai Lang Hu Bao” and the principles of hybrid algorithm mechanisms, we incorporated the leadership strategy of the head wolf and the hierarchical structure from GWO into the GJO algorithm. This integration serves to diversify the solutions during the algorithm’s iterations. By enhancing solution diversity, we have increased the GJO algorithm’s ability to escape local optima, thus reinforcing its global exploration capabilities.
2. Drawing inspiration from the collaborative mechanisms observed in natural populations, we introduced the Lagrange interpolation method to update the population’s positions within the GJO algorithm. This addition aims to enhance the algorithm’s convergence accuracy. The novel population updating mechanism strengthens the algorithm’s local exploitation capabilities.
3. We amalgamated the GWO algorithm, Lagrange interpolation method, and GJO algorithm to introduce the multi-strategy fusion GJO-GWO algorithm. Subsequently, we successfully integrated this algorithm with the KNN classifier to address feature selection problems.
4. We applied the proposed GJO-GWO algorithm to eight benchmark functions and ten feature selection problems. Experimental results indicate that, under identical experimental conditions, the GJO-GWO algorithm exhibits superior optimization performance, classification performance, and stability.

The organizational structure of this study is outlined as follows: In Section 2, we introduce the standard GJO algorithm. In Section 3, we present the multi-strategy fusion GJO-GWO algorithm. Section 4 explores the search and optimization performance of the GJO-GWO algorithm when dealing with complex benchmark functions. Section 5 investigates the feature selection method’s convergence and classification performance based on GJO-GWO. Finally, Section 6 summarizes the current research and discusses potential future research directions.

## 2. The GJO algorithm

The Golden Jackal Optimization (GJO) algorithm, developed by Chopra et al., draws inspiration from the biological population habits and predatory behavior of golden jackals. It is a novel metaheuristic algorithm that employs mathematical modeling techniques to simulate the hunting behavior of golden jackal populations, encompassing prey search, tracking, surrounding, and attacking processes. In the GJO algorithm, each individual within the population represents an initial feasible solution. The algorithm iteratively updates this population, simulating the golden jackal population’s search, tracking, surrounding, and attacking behavior until the pack successfully captures its prey, constituting the algorithm’s stopping condition. When this condition is met, it indicates no significant change between the previous and subsequent generations of the population, signifying the discovery of the optimal solution or optimal solution set. The GJO algorithm comprises four main processes.

### (1) Population initialization—Algorithm initialization

Like other metaheuristic algorithms, the initial population of the GJO algorithm is randomly distributed across the search space. It can be defined as:

$$Y_{0}=Y_{\min}+rand(Y_{\max}-Y_{\min})$$ (1)

Where *Y*_0_ represents the initial population of golden jackals. *Y*_max_ and *Y*_min_ correspond to the upper and lower boundaries of the search space, respectively. *rand* denotes a random number within the range of [0, 1].

In the GJO, the initial matrix of prey is defined as follows:

$$Prey=[\begin{array}{cccc} Y_{1,1} & Y_{1,2} & \cdots & Y_{1,d}\\ Y_{2,1} & Y_{2,1} & \cdots & Y_{2,d}\\ \vdots & \vdots & \vdots & \vdots \\ Y_{n,1} & Y_{n,2} & \cdots & Y_{n,d} \end{array}]$$ (2)

Where *Prey* represents the prey matrix, *y*_*i*,*j*_ represents the value of the *j*^th^ dimension for the *i*^th^ prey, *n* denotes the number of prey, and *d* represents the dimensionality of the problem being solved. During the algorithm’s iterative process, the fitness value of each prey is calculated using an appropriate fitness function. Therefore, the fitness values of all the prey can be expressed as follows:

$$F_{OA}=[\begin{array}{c} f(Y_{1,1};Y_{1,2};\cdots ;Y_{1,d})\\ f(Y_{2,1};Y_{2,1};\cdots ;Y_{2,d})\\ \vdots \\ f(Y_{n,1};Y_{n,2};\cdots ;Y_{n,d}) \end{array}]$$ (3)

Where *F*_*OA*_ is the fitness value matrix of all preys; *f* is the fitness function.

### (2) Searching and tracking the prey—iterative search process

Golden jackals exhibit inherent autonomous prey perception and tracking capabilities in the natural world. When a member of the population senses the presence of prey, the male jackal assumes the role of the leader, guiding the female jackal in the pursuit of the prey. This process can be represented through mathematical modeling as follows:

$$Y_{1}(t)=Y_{M}(t)-E\cdot |Y_{M}(t)-rl\cdot Prey(t)|$$ (4)

$$Y_{2}(t)=Y_{FM}(t)-E\cdot |Y_{FM}(t)-rl\cdot Prey(t)|$$ (5)

Where *t* represents the current iteration number. *Prey*(*t*) is the prey position at the *t*^th^ iteration. *Y*_*M*_(*t*) and *Y*_*FM*_(*t*) represent the positions of the male jackal and the female at the *t*^th^ iteration, respectively. *Y*_1_(*t*) and *Y*_2_(*t*) represent the updated positions of the male jackal and the female. *E* is the energy function of the prey avoiding the golden jackal is defined as:

$$E=E_{1}\cdot E_{0}$$ (6)

$$E_{0}=2r-1$$ (7)

$$E_{1}=c_{1}(1-(t/T))$$ (8)

Where *E*_1_ represents the energy decline process of the prey. *E*_0_ is the initial energy state of the prey. *r* is a random number between [0,1]. *c*_1_ is a constant 1.5. *T* represents the maximum number of iterations of the algorithm.

In Eq (4) and Eq (5), *rl* represents a random number generated from the Levy distribution, and it can be calculated using the following formula:

rl=0.05LF(y) (9)

Where LF is the Levy flight function, defined as:

$$LF(y)=0.01\times (\mu \times \sigma )/\left(\left|v^{\left(1/\beta \right)}\right|\right)$$ (10)

Where *μ* and *v* are random numbers between [0,1]. *β* = 1.5.

### (3) Surrounding and attacking the prey—iterative approximation process

As time elapses, the prey’s diminishing escape energy leads to a gradual encirclement and attack by the population of golden jackals. This process can be mathematically represented as follows:

$$Y_{1}(t)=Y_{M}(t)-E\cdot |rl\cdot Y_{M}(t)-Prey(t)|$$ (11)

$$Y_{2}(t)=Y_{FM}(t)-E\cdot |rl\cdot Y_{FM}(t)-Prey(t)|$$ (12)

### (4) Capturing the prey—algorithm termination

The population of golden jackals cooperatively surrounds and attacks the prey, eventually resulting in the successful capture of the prey. This process can be delineated as follows:

$$Y(t+1)=\frac{Y_{1}(t)+Y_{2}(t)}{2}$$ (13)

Where *Y*(*t*+1) is the position of the golden jackal at the (*t*+1)^th^ iteration. When *Y*_1_(*t*) and *Y*_2_(*t*) do not change significantly, that is, when *Y*(*t*+1) and *Y*(*t*) do not change significantly, the golden jackal successfully captures the prey, the algorithm iteration terminates, and the algorithm finds the optimal solution.

## 3. GJO-GWO hybrid optimization algorithm based on multi-strategy fusion

In the GJO algorithm, the individual search mechanism of the jackal serves as an efficient strategy for achieving rapid convergence. On the other hand, the collective behavior of the jackal ensures the algorithm’s capability to approach the global optimum. Therefore, striking a balance between these two strategies is paramount to facilitate the algorithm’s swift convergence toward the global optimal solution. Nevertheless, adhering to the No-Free-Lunch theorem [82], no solitary approach can comprehensively address all problems. To enhance the convergence and optimization performance of the GJO algorithm, this paper introduces the wolf search strategy from the GWO algorithm and integrates the Lagrange interpolation method.

### 3.1 A variation of GJO and GWO

#### 3.1.1 The GWO algorithm

The GWO is a classical metaheuristic algorithm that simulates the hunting behavior of a pack of grey wolves consisting of an *α* wolf, *β* wolf, *δ* wolf, and *ω* wolf. These wolves collaborate to search, track, and surround their prey. The algorithm is based on the mathematical model that emulates the hunting process of a grey wolf pack, aiming to optimize the objective function in the solution space iteratively. The primary model of the GWO algorithm is as follows:

*(1) Predation and hunting model of gray wolves*. In the GWO, wolves of different levels cooperate with each other and jointly search for prey. When the prey is found, *α* wolf leads the wolves of other levels to track, surround and attack the prey until the prey is captured. The process of the mathematical model is as follows:

$$D_{P}=|C_{P}X_{P}(t)-X(t)|$$ (14)

$$X_{P}(t+1)=X_{P}(t)-A_{P}\cdot D_{P}$$ (15)

Where *t* is the current iteration number. *p* = 1,2,3 represent *α* wolf, *β* wolf, and *δ* wolf. *X*_*P*_(*t*) and *X*(*t*) represent the positions of the prey and gray wolves at the *t*^th^ iteration. *D*_*p*_ represents the distance between *α* wolf, *β* wolf, and *δ* wolf and the prey in the *t*^th^ iteration. (*D*_*P*_→0 means that the gray wolves chase and attack and gradually surround and capture the prey). *A*_*P*_ and *C*_*P*_ are important parameters to control the hunting step of gray wolves.

*(2) The location update model of gray wolves*. In the process of hunting in a grey wolf pack, the position update equation for the wolves is defined as follows:

$$X(t+1)=\frac{X_{P}(t+1)}{3}=\frac{X_{1}+X_{2}+X_{3}}{3}$$ (16)

In this equation, *X*(*t*+1) represents the updated positions of the grey wolves after the *t*^th^ iteration, which corresponds to the initial positions of the grey wolves at the (*t*+1)^th^ iteration. The algorithm iterates until there is no significant change in the positions of the grey wolves between two consecutive iterations. This indicates that the grey wolves have successfully captured their prey, and the algorithm stops iterating.

#### 3.1.2 Introducing the *α*-wolf in the GJO algorithm

The integration of the Grey Wolf Optimization (GWO) algorithm into the GJO algorithm is motivated by the Chinese idiom: “Chai Lang Hu Bao.” As social animals such as jackals and wolves engage in cooperative hunting, their collaboration enables them to search and encircle prey across a more comprehensive spatial area. This collaborative effort compensates for individual differences, leading to an enhanced hunting success rate and an improved survival rate for the population. Even though both wolf and jackal packs collaborate in hunting, they exhibit distinctive hunting strategies. Jackal packs usually lack a strict hierarchical structure and rely on individual jackals forming groups to hunt collectively. In contrast, wolf packs are typically led by a dominant alpha wolf, adhering to a rigorous leadership hierarchy for coordinating group hunting activities.

To bolster the optimization performance of the GJO algorithm, this paper introduces the leadership strategy of the alpha wolf and the hierarchical structure concept from the GWO algorithm, disregarding competitive interactions between different populations. The alpha wolf is seamlessly integrated into the GJO algorithm as a secondary-tier individual, distinct from the golden jackals. The alpha Wolf’s primary role is to aid the golden jackals in their search for and encirclement of prey. This introduction of leadership strategy and hierarchical structure from the GWO algorithm is a deliberate effort to enhance the GJO algorithm’s optimization capability.

Introducing the alpha wolf makes notable adjustments to the critical iterative process of the Improved Golden Jackal Optimization (IGJO) algorithm. These specific modifications are detailed below.

*(1) Searching and tracking the prey—iterative search process*.
$$Y_{1}(t)=Y_{M}(t)-E\cdot |Y_{M}(t)-rl\cdot Prey(t)|$$ (17)
$$Y_{2}(t)=Y_{FM}(t)-E\cdot |Y_{FM}(t)-rl\cdot Prey(t)|$$ (18)
$$Y_{3}(t)=Y_{\alpha }(t)=Y_{\alpha }(t)-A\cdot |Y_{\alpha }(t)-C\cdot Prey(t)|$$ (19)
*(2) Surrounding and attacking the prey—iterative approximation process*.
$$Y_{1}(t)=Y_{M}(t)-E\cdot |rl\cdot Y_{M}(t)-Prey(t)|$$ (20)
$$Y_{2}(t)=Y_{FM}(t)-E\cdot |rl\cdot Y_{FM}(t)-Prey(t)|$$ (21)
$$Y_{3}(t)=Y_{\alpha }(t)=Y_{\alpha }(t)-A\cdot |C\cdot Y_{\alpha }(t)-Prey(t)|$$ (22)

Introducing the alpha wolf to the GJO algorithm instigates a transformation from the initial solitary hunting mechanism, reliant solely on the cooperation between golden jackals, to a collaborative hunting approach led by the golden jackal pair and the alpha wolf. As illustrated by Eqs (17) through (22), the inclusion of the alpha wolf extends the search radius of the initial population during the early stages (Eqs 17–19), consequently elevating the chances of detecting the prey. Additionally, it heightens the effectiveness of surrounding and capturing the prey (Eqs 20–22). The alpha wolf, guided by the golden jackal pair, significantly accelerates and enhances the encircling of the prey, reducing the probability of the prey escaping the population’s pursuit range. This, in turn, results in improved hunting speed and precision for the population.

However, with the introduction of the alpha wolf, the IGJO algorithm incorporates new parameters *A* and *C*, leading to an increase in algorithm complexity. To mitigate the potential impact of these new parameters, we substitute parameters *A* and *C* with parameters *E* and *rl* from the original GJO algorithm. As a result, Eq (19) and Eq (22) are modified as follows:

$$Y_{3}(t)=Y_{\alpha }(t)-E\cdot |Y_{\alpha }(t)-rl\cdot Prey(t)|$$ (23)

$$Y_{3}(t)=Y_{\alpha }(t)-E\cdot |rl\cdot Y_{\alpha }(t)-Prey(t)|$$ (24)

### 3.2 Collaborative updating mechanism of GJO-WOA based on Lagrange interpolation

Incorporating the hierarchical structure from the GWO algorithm and introducing the alpha wolf in the IGWO algorithm necessitate a modification of the original position update equation (Eq (13)) used in the basic GJO algorithm. In the fundamental GJO algorithm, when there is no alteration between the population positions of the previous generation and the current generation, signifying convergence, it implies that both the male and female jackals should occupy the same position. Combining Eq (13), it can be observed that *Y*_1_(*t*), *Y*_2_(*t*), and *Y*_3_(*t*) should be equal in this case. However, with the introduction of the hierarchical structure of the gray wolf pack and the *α* wolf, using Eq (13) as the position update equation does not consider the *α* wolf’s influence. This directly affects the precise application of the proposed improvement mechanism in Section 3.1 for enhancing the optimization performance of the GJO algorithm.

Considering the integration of the gray wolf optimization algorithm, the existing population will now consist of three fundamental elements: male jackals, female jackals, and *α* wolf. The positions of these three essential elements can be simplified as three points (*Y*_1_(*t*), *Y*_2_(*t*), and *Y*_3_(*t*)). To ensure the convergence of the position update equation, it is necessary to guarantee the intersection of the constructed three-point iteration formula. Additionally, considering the cooperation and convergence among the male jackals, female jackals, and *α* wolf, there should be interactions among the three points. Based on this, we introduce the Lagrange three-point interpolation formula, resulting in the new population update equation:

$$\begin{array}{l} Y(t+1)=\frac{\left(Y(t)-Y_{2}(t))(Y(t)-Y_{3}(t)\right)}{\left(Y_{1}(t)-Y_{2}(t))(Y_{1}(t)-Y_{3}(t)\right)}\cdot \frac{Y_{1}(t)}{3}+\frac{\left(Y(t)-Y_{1}(t))(Y(t)-Y_{3}(t)\right)}{\left(Y_{2}(t)-Y_{1}(t))(Y_{2}(t)-Y_{3}(t)\right)}\cdot \frac{Y_{2}(t)}{3}\\ \;+\frac{\left(Y(t)-Y_{1}(t))(Y(t)-Y_{2}(t)\right)}{\left(Y_{3}(t)-Y_{1}(t))(Y_{3}(t)-Y_{2}(t)\right)}\cdot \frac{Y_{3}(t)}{3} \end{array}$$ (25)

Where *Y*_1_(*t*), *Y*_2_(*t*), and *Y*_3_(*t*) represent the positions of the male jackal, the female jackal, and the *α* wolf in the *t*^th^ iteration, respectively. *Y*(*t*) represents the population position after the last iteration, that is, the initial position of the current population iteration. *Y*(*t*+1) represents the population position after the iterative update. The function of constant 3 is mainly used to control the convergence of the iterative equation and ensure that the distribution weights of male jackal, female jackal, and *α* wolf are consistent.

**Fig 1:**
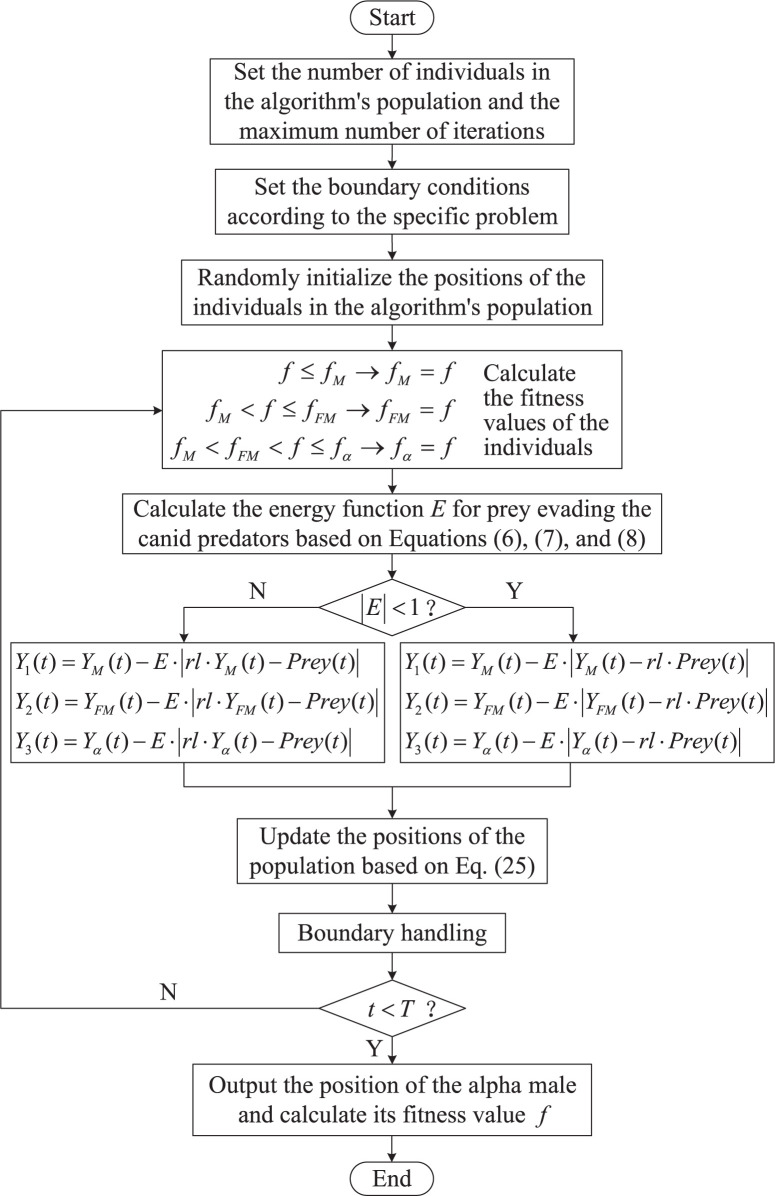
Flowchart of GJO-GWO algorithm.

Incorporating the enhancements detailed in Section 3.1 and Section 3.2, the improved iteration process of the Golden Jackal Optimization algorithm, referred to as the Multi-Strategy Integrated Golden Jackal-Grey Wolf Hybrid Optimization Algorithm (GJO-GWO), is illustrated in the basic flowchart presented in Fig 1.

### 3.3 GJO-GWO algorithm execution

Combining the algorithm improvement mechanism and Fig 1. The pseudocode for the proposed GJO-GWO algorithm is detailed in Algorithm 1.

**Algorithm 1: The GJO-GWO Algorithm**

Input: The population *N*, variable dimension *d*, and Maximum number of iterations *T*.

01: Initializing the population

02: **while** (*t*<*T*)

03:    **if** *f*≤*f*_*M*_

04:        *f*_*M*_ = *f*

05:    **elseif** *f*_*M*_<*f*≤*f*_*EM*_

06:        *f*_*EM*_ = *f*

07:    **elseif** *f*_*M*_<*f*_*FM*_<*f*≤*f*_*α*_

08:            *f*_*α*_ = *f*

09:    **end if**

10:    Calculating the random number *rl* associated with the *levy* function using Eqs (9) and (10)

11:    **for** (Iterating through each individual in the population)

12:            Computing the energy function *E* for prey avoiding the jackal wolves based on Eqs (6), (7), and (8)

13:            **if** |*E*|<1 (EXPLOITATION)

14:                $Y_{1}(t)=Y_{M}(t)-E\cdot |Y_{M}(t)-rl\cdot Prey(t)|$

15:                $Y_{2}(t)=Y_{FM}(t)-E\cdot |Y_{FM}(t)-rl\cdot Prey(t)|$

16:                $Y_{3}(t)=Y_{\alpha }(t)-E\cdot |Y_{\alpha }(t)-rl\cdot Prey(t)|$

17:            **else** (EXPLORATION)

18:                $Y_{1}(t)=Y_{M}(t)-E\cdot |rl\cdot Y_{M}(t)-Prey(t)|$

19:                $Y_{2}(t)=Y_{FM}(t)-E\cdot |rl\cdot Y_{FM}(t)-Prey(t)|$

20:                $Y_{3}(t)=Y_{\alpha }(t)-E\cdot |rl\cdot Y_{\alpha }(t)-Prey(t)|$

21:        **end if**

22:    Updating the population positions according to Eq (25)

23:      **end for**

24:      Boundary handling

25:      *t* = *t*+1

26: **end while**

Output: *Y*_1_(*t*) and *f*_*M*_

### 3.4 Computational complexity

This subsection primarily analyzes the time complexity and space complexity of the proposed GJO-GWO algorithm in this paper.

#### 3.4.1 Time complexity

Similar to other metaheuristic algorithms, the complexity of the GJO-GWO algorithm is predominantly influenced by three key processes: initialization, fitness evaluation, and individual updating [84, 85]. Notably, the complexity of fitness evaluation is intricately dependent on the intricacy of the specific optimization problem under consideration; consequently, we shall refrain from an exhaustive examination of this aspect. The GJO-GWO algorithm’s initialization process encompasses two distinct sub-processes: jackal initialization and wolf initialization. As a result, the initialization time complexity of the GJO-GWO algorithm stands at *O*(2*N*).

Further within the GJO-GWO algorithm, during the individual updating process, the male and female jackals and the wolf undergo updates subject to distinct constraint conditions. To elaborate, the individual update time complexity for the male and female jackals stands at O(T×N)+O(T×N×d). In contrast, the wolf’s individual update is characterized by a time complexity of *O*(*T*×*N*×*d*). Hence, the individual updating time complexity of the GJO-GWO algorithm amounts to O(T×N)+O(T×N×d)+O(T×N×d).

In summary, the overall time complexity of the GJO-GWO algorithm is summarized as O(N(T+2Td+2)). Where *N* denotes the population size, *T* represents the maximum number of iterations for the algorithm, and *d* signifies the dimensionality of the problem at hand.

#### 3.4.2 Space complexity

For the GJO-GWO algorithm, initializing the golden jackal and grey wolf populations occupies the most significant space. Therefore, the spatial complexity of the GJO-GWO algorithm can be characterized as *O*(2×*N*×*d*).

## 4. Test case 1: GJO-GWO for benchmark functions

In this section, we will comprehensively describe the experimental results of the GJO-GWO algorithm concerning benchmark functions. Through these experimental results, we will discuss the algorithm’s capabilities in finding optimal values and its convergence performance. Finally, we will employ two statistical tests, Wilcoxon and Friedman, to validate the statistical significance of the GJO-GWO algorithm’s superiority.

### 4.1 Experiment environment

#### (1) Environment

Operating system: 64-bit Windows 11.

CPU: 12th Gen Intel(R) Core(TM) i5-12500H 2.50 GHz Memory: 8G.

#### (2) Datasets

To validate the optimization performance of the GJO-GWO algorithm in solving complex functions, this study conducted numerical simulation experiments on eight benchmark functions. Detailed information on the datasets can be found in Table 2.

**Table 2:** Detailed information of benchmark functions.

Index	Function name	Unimodal/Multimodal	lb	ub
F1	Sum of Different Power	Unimodal	-100	100
F2	High Conditioned Elliptic	Unimodal	-100	100
F3	Powell Sum	Unimodal	-1	1
F4	Brown	Unimodal	-1	4
F5	Expanded Schaffer	Multimodal	-100	100
F6	HGBat	Multimodal	-100	100
F7	Periodic	Multimodal	-10	10
F8	Salomon	Multimodal	-100	100

#### (3) Parameter settings

This paper employed specific parameter settings for each algorithm to ensure fair and objective comparisons in the experimental setup. The parameter configurations for each algorithm are presented in Table 3.

**Table 3:** Parameter settings for algorithms.

Parameters	Value
Population number	30
Max_iteration	1000
Dim	30
Number of experiments	30
Comparison algorithms	AO [86], AOA [87], ASO [64], BOA [88], MVO [56], SSA [89], WOA [52], GWO [44], GJO [83], GJO-GWO
Evaluation metrics	Mean and standard deviation (Std)
Algorithm parameters	Algorithm parameters set the same as their original proposed values

### 4.2 Experimental results

#### 4.2.1 Convergence analysis

To conduct a preliminary analysis of the proposed GJO-GWO algorithm, we evaluated it on the eight benchmark functions presented in Table 2. The experimental results of the GJO-GWO algorithm and nine other metaheuristic algorithms are summarized in Table 4.

**Table 4:** Optimization results of different algorithms on benchmark functions.

Function	Index	AO	AOA	ASO	BOA	MVO	SSA	WOA	GWO	GJO	GJO-GWO
F1	Mean	4.82E-27	2.19E-25	8.31E+01	1.80E+50	9.09E+08	1.40E+11	1.80E-248	2.79E-215	0.00E+00	**0.00E+00**
	Std	2.64E-26	1.20E-24	2.21E+02	6.77E+50	1.16E+09	7.24E+11	0.00E+00	0.00E+00	0.00E+00	**0.00E+00**
F2	Mean	5.86E-207	1.97E-129	7.50E+03	1.83E-14	4.22E+06	2.80E+06	7.23E-168	4.33E-67	8.96E-124	**5.09E-316**
	Std	0.00E+00	1.08E-128	4.51E+03	1.09E-15	1.64E+06	1.94E+06	0.00E+00	1.28E-66	4.21E-123	**0.00E+00**
F3	Mean	8.22E-22	0.00E+00	8.19E-24	8.64E-17	7.12E-08	2.19E-07	2.05E-251	2.13E-230	0.00E+00	**0.00E+00**
	Std	4.50E-21	0.00E+00	2.82E-23	1.10E-16	3.69E-08	1.15E-07	0.00E+00	0.00E+00	0.00E+00	**0.00E+00**
F4	Mean	2.70E-199	1.71E+00	4.65E-21	1.46E-14	4.60E-04	2.30E-11	3.38E-175	1.59E-72	1.46E-130	**1.59E-320**
	Std	0.00E+00	1.01E+00	6.64E-21	1.42E-15	1.28E-04	3.91E-12	0.00E+00	6.79E-72	6.51E-130	**0.00E+00**
F5	Mean	0.00E+00	0.00E+00	9.82E+00	1.07E+01	1.15E+01	8.66E+00	1.47E+00	5.02E+00	9.78E-01	**0.00E+00**
	Std	0.00E+00	0.00E+00	6.68E-01	5.64E-01	6.68E-01	1.07E+00	2.95E+00	1.58E+00	2.31E+00	**0.00E+00**
F6	Mean	4.40E-01	4.88E-01	4.51E-01	4.69E-01	7.55E-01	6.18E-01	**3.79E-01**	4.40E-01	4.59E-01	5.00E-01
	Std	9.41E-02	1.05E-02	5.60E-02	2.63E-02	4.52E-01	3.38E-01	9.57E-02	4.77E-02	3.44E-02	**2.32E-03**
F7	Mean	9.00E-01	9.00E-01	1.00E+00	7.41E+00	1.00E+00	1.00E+00	1.00E+00	1.28E+00	9.29E-01	**9.00E-01**
	Std	4.52E-16	4.52E-16	5.83E-17	4.55E-01	3.95E-04	1.72E-11	1.36E-01	3.27E-01	1.19E-01	**4.52E-16**
F8	Mean	2.84E-05	9.99E-02	8.57E-01	3.05E-01	5.73E-01	7.43E-01	1.37E-01	1.43E-01	9.99E-02	**7.44E-162**
	Std	1.56E-04	1.42E-09	1.22E-01	9.55E-03	9.80E-02	1.17E-01	8.50E-02	5.04E-02	3.23E-10	**8.32E-162**

Table 4 illustrates the results obtained under identical experimental conditions:

1. Overall Optimization Results: The GJO-GWO algorithm consistently demonstrates smaller mean values and standard deviations than the other algorithms across most benchmark functions. This signifies that the enhanced algorithm exhibits superior convergence performance and optimization capabilities compared to its counterparts.
2. Optimization Results for Unimodal Functions (F1-F4): GJO-GWO consistently achieves smaller mean values and standard deviations than the nine other algorithms across the four single-peak functions. This is evidence of the algorithm’s exceptional optimization performance and stability in locating global optima. These findings underscore GJO-GWO’s enhanced local exploration capabilities on unimodal functions.
3. Optimization Results for Multimodal Functions (F5-F8): Except for a slightly lower performance on F6, GJO-GWO consistently outperforms the other algorithms when optimizing multi-peak functions. Consequently, concerning the overall optimization results for multimodal functions, GJO-GWO exhibits superior global exploration capabilities compared to its counterparts.

The experimental results unequivocally establish that the GJO-GWO algorithm achieves convergence in solving complex functions. It consistently outperforms the competing algorithms regarding mean values and standard deviations, thus highlighting its robust optimization and exploration capabilities. These outcomes validate the efficacy of the GJO-GWO algorithm in tackling function optimization tasks.

**Fig 2:**
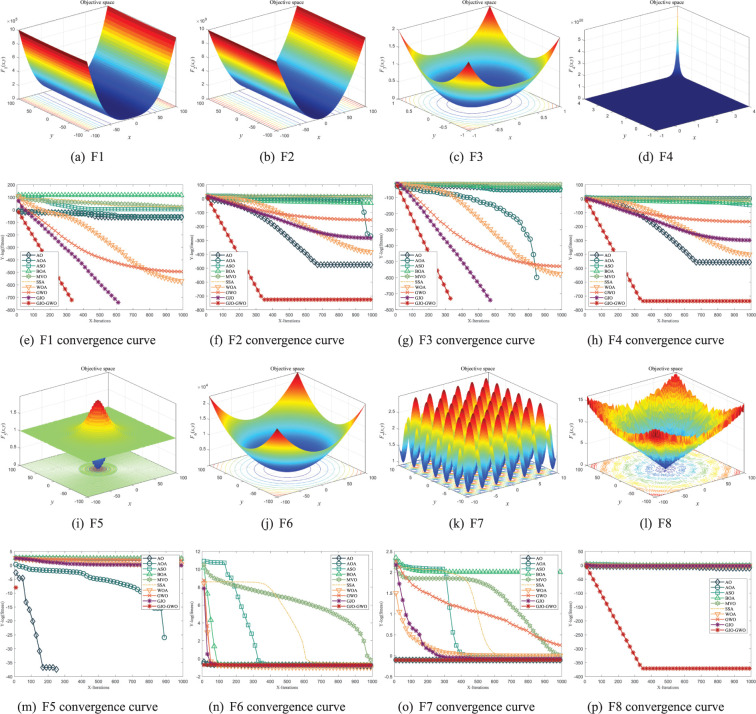
Benchmark function graphs and convergence curves of different algorithms.

To visually compare and analyze the superior performance of the GJO-GWO algorithm in solving complex functions in comparison to the other nine metaheuristic algorithms, we have plotted basic graphs of the eight benchmark functions and convergence curves for each algorithm after 1000 iterations, as depicted in Fig 2.

As observed in Fig 2, it becomes evident that the GJO-GWO algorithm exhibits the fastest and earliest convergence for both unimodal and multimodal functions. This observation strongly suggests that the GJO-GWO algorithm possesses a higher convergence rate and superior convergence accuracy, thus affirming its exceptional optimization performance in addressing complex functions.

In summary, under uniform experimental conditions, the GJO-GWO algorithm, which combines multiple strategies from the Golden Jackal Optimization and Grey Wolf Optimization, surpasses the other nine metaheuristic algorithms in terms of both mean and standard deviation indicators. This substantiates GJO-GWO’s superior local exploitation capability and enhanced global exploration capability in solving unimodal and multimodal functions. Furthermore, the minimal standard deviation indicates GJO-GWO’s heightened robustness when optimizing complex functions.

#### 4.2.2 Statistical test analysis

To comprehensively and objectively assess the optimization performance of the GJO-GWO algorithm, this study employed two statistical tests for evaluation.

Firstly, to assess the significant differences between the GJO-GWO algorithm and the other algorithms, pairwise comparisons were conducted using the GJO-GWO algorithm as the control. The Wilcoxon [90] rank-sum test was performed at a significance level of 5%, and the corresponding *p*-values are presented in Table 5. In Table 5, the symbols ‘+’ and ‘-’ indicate whether the algorithm has a significant statistical significance advantage (‘+’) or not (‘-’). The results in Table 5 reveal that the *p*-values obtained from the Wilcoxon rank-sum test for the GJO-GWO algorithm against all the other algorithms are significantly smaller than 0.05. This signifies that the GJO-GWO algorithm demonstrates a noteworthy advantage over the nine compared algorithms regarding optimization performance.

**Table 5:** Wilcoxon rank sum test results.

Function	Vs. AO	Vs. AOA	Vs. ASO	Vs. BOA	Vs. MVO	Vs. SSA	Vs. WOA	Vs. GWO	Vs. GJO
F1	2.45E-314	1.25E-315	0.00E+00	0.00E+00	3.72E-310	0.00E+00	6.22E-204	5.31E-170	2.17E-34
F2	2.56E-184	1.20E-318	0.00E+00	1.90E-315	3.89E-318	0.00E+00	8.20E-239	1.54E-269	2.19E-235
F3	0.00E+00	7.05E-176	0.00E+00	0.00E+00	1.85E-306	0.00E+00	4.43E-202	1.86E-159	4.69E-26
F4	7.59E-199	0.00E+00	0.00E+00	0.00E+00	2.06E-311	0.00E+00	2.07E-238	1.23E-271	6.92E-236
F5	2.92E-51	1.68E-301	0.00E+00	0.00E+00	2.48E-312	0.00E+00	0.00E+00	0.00E+00	0.00E+00
F6	6.24E-74	4.45E-250	6.50E-22	3.54E-208	2.28E-310	0.00E+00	1.67E-255	1.09E-200	1.77E-228
F7	1.21E-03	1.66E-259	0.00E+00	0.00E+00	1.29E-251	0.00E+00	0.00E+00	0.00E+00	0.00E+00
F8	0.00E+00	0.00E+00	0.00E+00	0.00E+00	2.28E-308	0.00E+00	0.00E+00	0.00E+00	0.00E+00
+/-	8/0	8/0	8/0	8/0	8/0	8/0	8/0	8/0	8/0

Secondly, while the Wilcoxon rank-sum test primarily focuses on comparing the performance between two algorithms, it is necessary to effectively evaluate the performance of each algorithm within the entire set. As a non-parametric test, we employed the Friedman test [91] to determine whether there were significant differences among multiple algorithm distributions. This test utilizes ranks to assess the overall optimization performance of the GJO-GWO algorithm across the eight benchmark functions and identify significant differences among various observed data. The results of the Friedman test for the GJO-GWO algorithm are presented in Table 6. As shown in Table 6, the GJO-GWO algorithm achieves the highest rank among the ten algorithms, thus confirming its significant advantage over the nine compared metaheuristic algorithms.

**Table 6:** Friedman test results.

Algorithm	Friedman rank test	Rank
GJO-GWO	2.3125	1
GJO	3.5625	3
GWO	5.5000	6
AO	3.0000	2
AOA	4.6250	5
ASO	6.7500	7
BOA	8.0000	8
MVO	8.8750	10
SSA	8.3750	9
WOA	4.0000	4

In conclusion, under uniform constraint conditions, the GJO-GWO algorithm exhibits superior overall metrics (lower mean and standard deviation) and statistical metrics (lower Wilcoxon rank-sum test and Friedman test results) compared to the nine algorithms. These findings underscore the GJO-GWO algorithm’s enhanced local exploitation and global exploration capabilities.

### 4.3 Discussion

In Case 1, we conducted both convergence analysis and statistical tests to evaluate the performance of the GJO-GWO algorithm when solving benchmark functions of varying modes. By studying the experimental results of ten metaheuristic algorithms across different function modes, we comprehensively understood how the GJO-GWO algorithm performs in optimizing problems.

The GJO-GWO algorithm performs better in finding optimal values, stability, convergence, and statistical significance. These remarkable achievements can be attributed to the introducing of the alpha wolf and the cooperative strategies among the alpha wolf, male jackal, and female jackal. Primarily, during the initial iterations of the algorithm, the introduction of the alpha wolf expands the algorithm’s search space, increasing the likelihood of the population discovering prey and thereby enhancing the algorithm’s chances of escaping local optima. Furthermore, in the later iterations of the algorithm, the cooperation between the alpha wolf, male jackal, and female jackal accelerates the population’s updating process, facilitating the algorithm in converging to the global optimum more rapidly.

However, it should be noted that as a hybrid optimization algorithm, the GJO-GWO algorithm exhibits a significant increase in both time and space complexity (as detailed in Section 3.4). This implies that while the proposed algorithm enhances performance, it also demands higher computational resources. Nevertheless, we consider this performance improvement worthwhile because, within our acceptable limits, we are willing to make certain computational sacrifices to achieve superior optimization performance. Hence, we regard the GJO-GWO algorithm as a meaningful algorithm enhancement.

## 5. Test case 2: GJO-GWO for feature selection

This section will provide a detailed description of applying the GJO-GWO algorithm to feature selection problems. Firstly, we will outline the specific implementation process of the GJO-GWO algorithm in the context of feature selection. Secondly, we will assess the performance of the GJO-GWO algorithm in feature selection problems based on experimental results. Finally, we will employ statistical analysis methods to confirm the exceptional performance of the GJO-GWO algorithm in feature selection tasks.

### 5.1 Implementation process

#### 5.1.1 Initialization

1. *Binary Conversion*. During the initialization phase, the GJO-GWO algorithm randomly generates an initial population of *N* candidate solutions, where each individual represents a feature subset to be evaluated. However, feature selection problems are typically binary discrete problems. Therefore, when using the GJO-GWO algorithm to select feature subsets for evaluation, it is necessary to map the feature vectors from continuous to binary discrete space. This transformation is defined as:
$$x_{binary}=\{\begin{array}{ll} 0, & x_{ij}\leq 0.5\\ 1, & x_{ij}>0.5 \end{array}$$ (26)
  Where *x*_*binary*_ represents the feature value after binarization. *x*_*ij*_ indicates the real value and *i* = *j* = 1,2,⋯,*N*, *j* = 1,2,⋯,*D*.
2. *Fitness function calculation*. Once the feature subsets are selected, it is necessary to calculate the fitness function for these feature subsets to determine their quality. The equation for computing the fitness function is defined as:
$$Fitness=\alpha \gamma_{R}(D)+\beta \frac{\left|R\right|}{\left|C\right|}$$ (27)
  Where *γ*_*R*_(*D*) represents the KNN classification error rate. |*R*| represents the length of the selected feature subset. |*C*| represents the total number of features in the datasets. *α*∈(0,1) represents the importance of classification quality, and *β* = (1−*α*) represents the importance of the subset length [2].

#### 5.1.2 Updating solutions

Solution updating is a crucial component of the optimization algorithm for feature selection problems, and different algorithms employ various strategies. In this critical step, the GJO-GWO algorithm continually adjusts each selected solution using Eqs (17) through (24) to pursue improved solutions. Then, through Eq (27), the fitness evaluation of the new generation of feature subsets is performed to determine the best feature combinations. Typically, this process requires multiple iterations until the termination criteria are met. In this research, the termination criteria usually refer to reaching the maximum number of iterations, which helps evaluate the performance level of the GJO-GWO algorithm.

#### 5.1.3 Classification

As a typical wrapper feature selection method, the feature selection approach based on GJO-GWO not only employs GJO-GWO to search for feature subsets but also requires combining a learning algorithm to simultaneously evaluate these subsets, ensuring that while reducing the number of features, a high classification accuracy is maintained. In this study, we utilized a KNN classifier (*k* = 5) as the learning algorithm to evaluate the feature subsets selected by the GJO-GWO algorithm. We adopted the hold-out method to classify the original dataset, randomly splitting it into two portions: 80% as the training set and 20% as the test set. The KNN classifier (*k* = 5) assessed the classification accuracy.

### 5.2 Experimental evaluation

In this section, we present the experimental results and discuss the performance of the proposed feature selection method based on the GJO-GWO algorithm. To achieve this, a set of ten UCI [92] classification datasets with multiple features and redundant information was selected for analysis under the same constraints.

#### 5.2.1 Experiment environment

*(1) Datasets*. To validate the effectiveness of applying the GJO-GWO algorithm to feature selection problems, numerical experiments were conducted on ten datasets from the UCI repository [92]. Detailed information about these datasets is presented in Table 7.

**Table 7:** Detailed information of datasets.

Index	Datasets	Features	Instances	Classes
D1	Carcinom	9182	174	11
D2	colon	2000	62	2
D3	GLIOMA	4434	50	4
D4	leukemia	7070	72	2
D5	lung	3312	203	5
D6	lymphoma	4026	96	9
D7	orlraws10P	10304	100	10
D8	pixraw10P	10000	100	10
D9	Prostate-GE	5966	102	2
D10	USPS	256	9298	10

*(2) Parameter settings*. To provide a comprehensive and objective validation of the superiority and feasibility of the feature selection method based on GJO-GWO, the parameter settings for each algorithm are presented in Table 8.

**Table 8:** Parameter settings for algorithms.

Parameters	Value
Population number	10
Max iterations	100
Dim	Number of features
K-neighbor	5
Number of experiments	10
Comparison algorithms	ABC [39], ASO [63], BA [93], DE [94], GSA [95], MVO [55], PSO [96], SSA [58], GWO [42], EO [97], HHO [98], MPA [99], GJO, GJO-GWO
ASO parameters	*α* = 50, *β* = 0.2
BOA parameters	*c* = 0.01, *p* = 0.8
BA parameters	*α* = 0.9, *γ* = 0.9
GA parameters	*CR* = 0.8, *MR* = 0.01
GSA parameters	*G*_0_ = 100, *α* = 20
DE parameters	*CR* = 0.9
PSO parameters	*c*_1_ = *c*_2_ = 2, *ω* = 0.9

Table 8 displays the parameter configurations for the comparative feature selection algorithms used in the experiments. These parameters encompass the population size, maximum number of iterations, and other algorithm-specific settings.

#### 5.2.2 Evaluation metrics

To comprehensively evaluate the GJO-GWO algorithm’s performance in feature selection problems, we utilized the metrics listed in Table 9 to assess the algorithm [100].

**Table 9:** Evaluation metrics of the models’ performance.

Metrics	Value
Accuracy	$Acc=\frac{TP+TN}{TP+TN+FP+FN}$
Mean Accuracy	$\bar{Acc}=\frac{1}{10}\sum_{i=1}^{10}Acc(i),i=1,2,\cdots ,10$
Mean Number	$\bar{Num}=\frac{1}{10}\sum_{i=1}^{10}Fea(i),i=1,2,\cdots ,10$
Mean Runtime	$\bar{Rt}=\frac{1}{10}\sum_{i=1}^{10}Runtime(i),i=1,2,\cdots ,10$

In Table 9, Accuracy represents the classification accuracy of the algorithm on each dataset. *TP*, *TN*, *FP*, and *FN* refer to the true positive, true negative, false positive, and false. negative. $\bar{Acc}$ represents the average classification accuracy across datasets. A higher average classification accuracy indicates better classification performance of the algorithm. $\bar{Num}$ represents the algorithm’s average number of selected features across datasets. A lower average number of selected features indicates a more significant reduction in redundant information. $\bar{Rt}$ represents the average runtime of the algorithm across datasets. A smaller average runtime implies faster optimization performance. *Acc*(*i*) represents the classification accuracy in the *i*^th^ experiment. *Fea*(*i*) represents the number of selected features in the *i*^th^ experiment. *Runtime*(*i*) is the runtime in the *i*^th^ experiment, where *i* ranges from 1 to 10, denoting the ten repeated experiments.

#### 5.2.3 Experimental results

**Fig 3:**
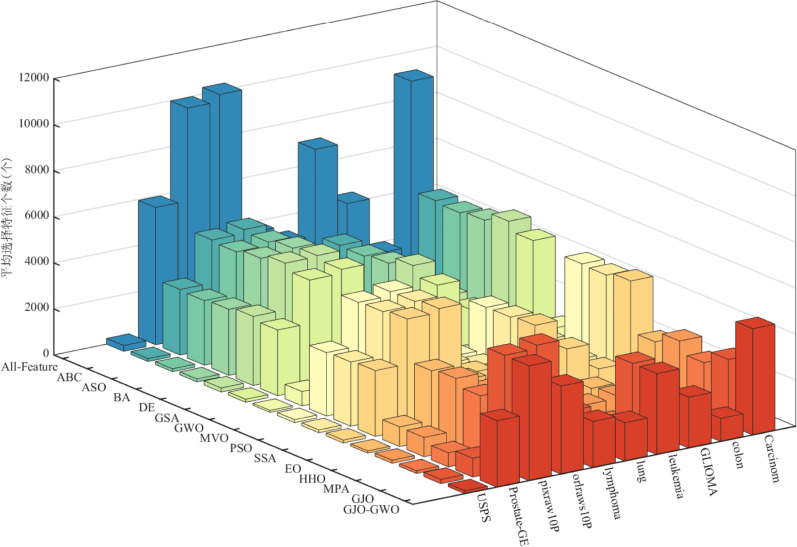
Average number of selected features.

**Fig 4:**
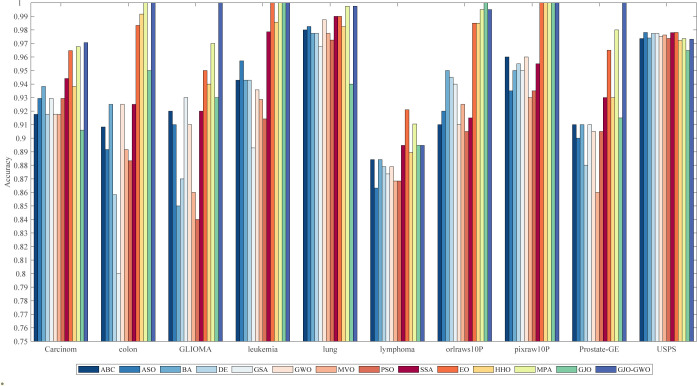
Bar chart of average classification accuracy for different algorithms.

Based on the parameter settings in Table 8, we conducted numerical experiments to compare the performance of the feature selection method based on GJO-GWO with ten other metaheuristic algorithms on the ten classification datasets listed in Table 7. The experimental results are presented in Tables 10 and 11. Tables 10 and 11 display the average number of selected features and average classification accuracy of GJO-GWO when used for feature selection compared to the 13 other algorithms on the ten classification datasets, all with *k* = 5. The best-performing values in the tables have been highlighted in bold text. Additionally, Figs 3 and 4 visually represent each algorithm’s average number of selected features and average classification accuracy.

**Table 10:** Average number of selected features.

Index	D1	D2	D3	D4	D5	D6	D7	D8	D9	D10
Allfeatures	9182.0	2000.0	4434.0	7070.0	3312.0	4026.0	10304.0	10000.0	5966.0	256.0
ABC	4433.0	907.9	2079.5	3340.3	1542.3	1874.2	4880.4	4713.7	2844.4	127.5
ASO	4345.9	897.6	2007.1	3315.0	1529.0	1811.1	4778.0	4637.2	2803.2	126.5
BA	4465.9	937.5	2087.1	3447.8	1560.0	1919.7	4957.4	4798.7	2866.5	125.8
DE	4863.1	1052.8	2184.4	3791.6	1804.0	2051.2	5075.9	5029.2	3000.8	177.5
GSA	4469.1	917.7	2062.6	3382.1	1568.8	1865.0	4921.8	4741.0	2853.8	130.1
MVO	4462.5	910.3	2070.3	3380.2	1540.4	1869.5	4978.2	4779.4	2837.2	131.6
PSO	4340.0	885.4	2008.5	3298.5	1481.0	1834.7	4834.6	4653.9	2756.3	125.7
SSA	4296.4	884.9	2020.8	3319.6	1513.6	1871.8	4852.9	4694.0	2789.7	124.4
GWO	4481.4	947.8	2097.0	3408.1	1574.0	1923.6	4985.6	4816.2	2857.0	126.3
EO	2277.9	224.4	825.6	543.7	162.4	541.6	2331.3	3011.7	857.4	**95.5**
HHO	2757.5	228.7	960.6	820.4	249.3	585.6	2339.3	3118.6	842.1	133.5
MPA	**2257.0**	**206.4**	**509.1**	**517.1**	**126.3**	**534.6**	**2024.7**	**2805.2**	**614.3**	114.5
GJO	2839.2	593.1	1774.4	3520.2	371.6	2015.8	5162.2	5009.4	818.0	184.0
GJO-GWO	4619.4	1005.9	2212.5	3520.0	1674.3	2006.8	3830.3	4991.6	2891.8	133.2

**Table 11:** Average classification accuracy of different algorithms on datasets.

Index	D1	D2	D3	D4	D5	D6	D7	D8	D9	D10
ABC	0.9176	0.9083	0.9200	0.9429	0.9800	0.8842	0.9100	0.9600	0.9100	0.9736
ASO	0.9294	0.8917	0.9100	0.9571	0.9825	0.8632	0.9200	0.9350	0.9000	0.9781
BA	0.9382	0.9250	0.8500	0.9429	0.9775	0.8842	0.9500	0.9500	0.9100	0.9740
DE	0.9176	0.8583	0.8700	0.9429	0.9775	0.8789	0.9450	0.9550	0.8800	0.9774
GSA	0.9294	0.8000	0.9300	0.8929	0.9675	0.8737	0.9400	0.9500	0.9100	0.9774
MVO	0.9176	0.9250	0.9100	0.9357	0.9875	0.8789	0.9100	0.9600	0.9050	0.9751
PSO	0.9176	0.8917	0.8600	0.9286	0.9775	0.8684	0.9250	0.9300	0.8600	0.9762
SSA	0.9294	0.8833	0.8400	0.9143	0.9725	0.8684	0.9050	0.9350	0.9050	0.9737
GWO	0.9441	0.9250	0.9200	0.9786	0.9900	0.8947	0.9150	0.9550	0.9300	0.9779
EO	0.9647	0.9833	0.9500	1.0000	0.9900	**0.9211**	0.9850	1.0000	0.9650	**0.9782**
HHO	0.9382	0.9917	0.9400	0.9857	0.9825	0.8895	0.9850	1.0000	0.9300	0.9722
MPA	0.9676	1.0000	0.9700	1.0000	0.9975	0.9105	0.9950	1.0000	0.9800	0.9735
GJO	0.9059	0.9500	0.9300	1.0000	0.9400	0.8947	**1.0000**	1.0000	0.9150	0.9648
GJO-GWO	**0.9706**	**1.0000**	**1.0000**	**1.0000**	**0.9975**	0.8947	0.9950	**1.0000**	**1.0000**	0.9731

*(1) Impact of a single indicator on algorithm performance*. Regarding the average number of selected features, as indicated by Table 10 and Fig 3, the feature selection method based on MPA demonstrates superior overall performance. Conversely, the performance of the feature selection method based on GJO-GWO is relatively weaker than the other 13 contrast algorithms on most datasets. However, it reduces the number of selected features by half relative to the total number.

Regarding the average classification accuracy indicator, as shown in Table 11 and Fig 4, the feature selection method based on GJO-GWO achieves the best classification accuracy among all 14 algorithms on eight datasets, ranking first overall. Moreover, it attains 100% accuracy on five datasets (D2, D3, D4, D8, and D9).

*(2) Impact of multiple indicators on algorithm performance*. When considering both the average number of selected features and the average classification accuracy, the feature selection method based on GJO-GWO exhibits suboptimal performance in terms of the average number of selected features but demonstrates superior performance in classification accuracy. This aligns well with the goal of feature selection, which is to balance the selection of features while ensuring high classification accuracy. Therefore, considering the combined influence of these two metrics, the feature selection method based on GJO-GWO outperforms the other 14 algorithms.

In summary, if one only considers the impact of a single metric on the proposed algorithm in this paper, the performance of the feature selection method based on GJO-GWO is moderate. However, when comprehensively considering the interplay between the two metrics, the performance of the proposed algorithm stands out as optimal. The inconsistency in the conclusion arises from the fact that even if the algorithm identifies fewer features through the search, it does not necessarily translate to higher classification accuracy, let alone superior algorithm performance. This highlights that being locally optimal at every step does not guarantee global optimality. The algorithm can be deemed superior performance only by selecting an appropriate number of features and ensuring optimal classification accuracy.

At first glance, the objective of the feature selection problem may seem straightforward: minimize the number of selected features while maximizing classification accuracy. However, when we consider storage space constraints, the motivation behind choosing fewer features becomes clear—it is to ensure faster algorithm execution within the same experimental environment. Consequently, in the context of solving the feature selection problem, the number of selected features is intimately linked with an algorithm’s runtime. Therefore, scrutinizing the specific runtime of algorithms in solving the feature selection problem becomes paramount for performance assessment.

**Fig 5:**
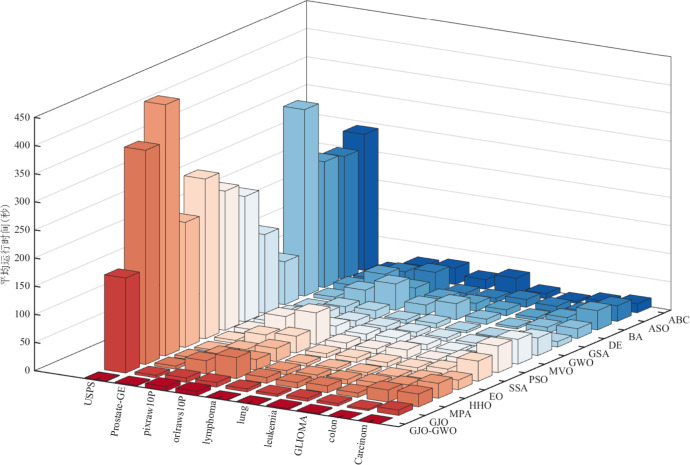
Average runtime of different algorithms.

In this context, to validate that the GJO-GWO-based feature selection method achieves superior classification accuracy and exhibits faster runtime, we meticulously recorded the average runtime of each algorithm across ten experiments on the ten classification datasets. This data is presented in detail in Table 12, with the best-performing values highlighted in bold for clarity. We provide a visual representation of the average runtime of each algorithm in Fig 5.

**Table 12:** Average runtime of different algorithms.

Index	D1	D2	D3	D4	D5	D6	D7	D8	D9	D10
ABC	15.8964	11.2883	3.5635	8.8650	27.9624	17.1204	29.2105	24.5929	7.1001	241.8138
ASO	27.7699	9.3713	10.0063	13.6611	7.7309	8.0072	36.8482	29.3979	8.6546	217.8057
BA	34.2909	13.4094	5.0749	10.6288	13.6085	14.9403	24.1208	40.2080	11.7360	223.5148
DE	17.3292	11.3482	3.6586	9.1551	28.3423	17.5531	46.5898	27.1963	6.6174	331.5218
GSA	9.5096	4.2992	**1.7572**	3.9998	8.4054	6.3164	14.6488	13.0252	2.8379	76.3209
MVO	31.2839	10.4833	4.8142	6.4164	8.0711	9.7012	11.4649	17.2510	6.4677	139.8658
PSO	43.9523	11.4441	6.4590	9.5972	12.5643	11.3051	17.1917	27.9966	8.2329	222.6997
SSA	47.5240	15.8022	6.1817	15.3024	8.5662	10.6958	56.0515	41.1383	8.5792	247.3879
GWO	35.7137	13.0713	7.1943	8.0302	7.8525	7.0885	29.6850	23.6272	5.6213	284.1789
EO	17.2340	10.6020	6.7263	5.3530	4.8641	4.9371	23.3635	12.6128	3.0602	222.3281
HHO	26.5467	15.4235	10.5478	8.2655	7.8866	7.3512	18.8006	18.5272	4.4308	446.5867
MPA	22.8785	21.5587	7.9566	12.2473	9.6604	10.3295	37.3628	23.2741	5.4276	380.7994
GJO	9.5252	2.7638	4.3127	5.2719	4.2228	5.4279	8.2307	8.0602	4.3155	169.0241
GJO-GWO	**0.5665**	**0.1417**	2.2291	**2.6694**	**0.7604**	**1.1737**	**7.9078**	**7.8233**	**1.9089**	**1.0543**

Combined with the insights from Table 12 and the visual representation in Fig 5, it becomes evident that the feature selection method utilizing GJO-GWO boasts a significantly reduced runtime compared to the comparative algorithms across all ten datasets. Remarkably, even as dataset sizes grow (measured by the number of features multiplied by the number of instances), the algorithm’s runtime remains notably lower than that of the comparative algorithms. Considering the findings from Table 11 and Fig 4, it is clear that the proposed feature selection method based on GJO-GWO achieves enhanced computational efficiency and superior classification accuracy.

In conclusion, by considering the collective impact of three pivotal indicators: average selected feature count, average classification accuracy, and average runtime, it is evident that the feature selection method based on GJO-GWO excels in addressing the feature selection problem.

#### 5.2.4 Convergence curve analysis

**Fig 6:**
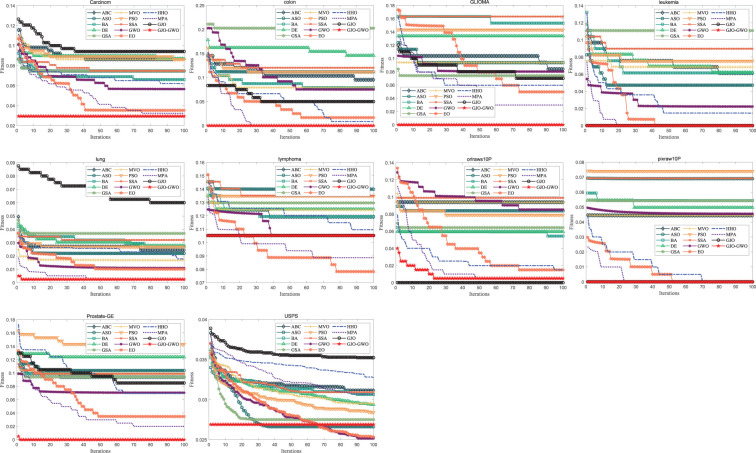
Convergence curves of the different algorithms on the feature selection.

The convergence curve depicts the trend of a turn at a particular point or interval, offering insights into the convergence and stability of an algorithm during the optimization process. Therefore, this section strongly emphasizes conducting a detailed analysis of the convergence curves. Fig 6 presents the convergence curves of 13 metaheuristic algorithms for feature selection. By examining the convergence curves in Fig 6, it becomes evident that except for D6, D7, and D10, the GJO-GWO algorithm achieves faster convergence to the optimal solution on the other datasets. This observation underscores that the GJO-GWO algorithm excels in terms of convergence speed and accuracy in most cases, further highlighting its outstanding performance in optimization.

#### 5.2.5 Statistical analysis

To comprehensively evaluate the GJO-GWO-based feature selection method’s performance concerning three key metrics: average selected feature count, classification accuracy, and runtime, we have employed the following approach:

**1. Comprehensive Ranking:** We initiated the analysis by computing comprehensive rankings for various algorithms across these three metrics based on data from Tables 10–12. The summarized rankings are presented in Table 13.

**Table 13:** Comprehensive rankings of different algorithms in three indicators.

Index	Rankings of average selected feature count	Rankings of average classification accuracy	Rankings of average running time
ABC	8	8	7
ASO	7	9	9
BA	11	7	13
DE	14	11	11
GSA	10	12	3
MVO	1	10	5
PSO	5	13	10
SSA	6	14	14
GWO	12	5	8
EO	3	3	4
HHO	4	4	6
MPA	2	2	12
GJO	9	6	2
GJO-GWO	13	1	1

**Fig 7:**
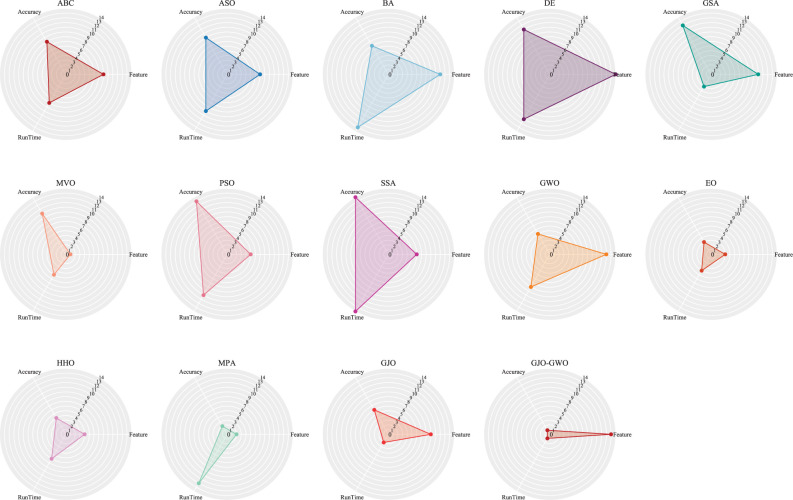
The three-indicator ranking radar chart of different algorithms.

**2. Radar Chart Visualization:** Subsequently, we used the rankings from Table 13 to create radar charts, exemplified in Fig 7. These radar charts visually depict algorithmic rankings across the performance metrics, with a smaller enclosed area indicating superior performance.

Table 13 and Fig 7 are vital tools for assessing algorithmic performance based on average selected feature count, classification accuracy, and runtime. Fig 7, the radar chart, is crafted to visually represent the ranking outcomes from Table 13. A smaller enclosed area within the radar chart signifies superior algorithmic performance.

Fig 7 visually portrays algorithm rankings across the three essential performance indicators. The radar chart, as displayed in Fig 7, is constructed using the complete ranking data from Table 13. When considering the consistent weighting of three indicators on the algorithm’s performance, the triangle area formed by the feature selection method based on EO is smaller than the other 13 comparative algorithms, and the feature selection method based on GJO-GWO ranks third. However, when considering average classification accuracy and average runtime as the primary influencing factors, the performance of the feature selection method based on GJO-GWO is optimal. Regarding the average number of selected features, the MVO algorithm exhibits the best performance but at the cost of reducing average classification accuracy and average runtime.

**Fig 8:**
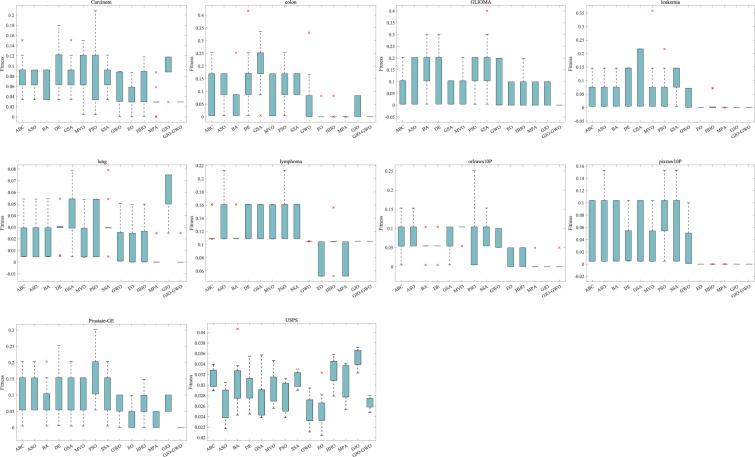
Boxplot of optimal fitness for 10 independent experiments of different algorithms.

**3. Optimal Fitness Boxplots:** We conducted ten independent experiments for each algorithm to record the optimal fitness values in solving the feature selection problem. These values are showcased in boxplots in Fig 8.

Fig 8 depicts the box plots of the optimal fitness values obtained by different algorithms in 10 independent experiments. From Fig 8, it can be observed that the feature selection method based on GJO-GWO exhibits a distribution of optimal fitness values in a favorable and narrow range across all ten datasets. This indicates that the improved algorithm possesses better search performance and demonstrates superior stability in finding optimal feature subsets.

In summary, considering the combined impact of the three metrics on algorithm performance, the higher average classification accuracy, shorter average running time, and more reasonable selection of feature subsets validate that the feature selection method based on GJO-GWO not only achieves faster search but also demonstrates more robust stability in solving feature selection problems.

**4. Wilcoxon rank-sum test:** To better analyze the superiority of the GJO-GWO algorithm in feature selection, we recorded the average fitness of each algorithm for feature selection tasks. Subsequently, a rank-sum test was conducted for statistical analysis, and the results are presented in Table 14.

**Table 14:** Wilcoxon rank sum test results.

Index	D1	D2	D3	D4	D5	D6	D7	D8	D9	D10	+/-
Vs. ABC	3.53E-18	3.74E-18	3.80E-18	3.74E-18	3.79E-18	3.75E-18	3.74E-18	3.11E-18	3.73E-18	3.64E-18	10/0
Vs. ASO	9.12E-19	5.68E-19	8.35E-19	7.63E-19	6.94E-19	6.94E-19	7.63E-19	8.36E-19	9.91E-19	**7.44E-01**	9/1
Vs. BA	3.73E-18	3.67E-18	3.80E-18	3.69E-18	3.80E-18	3.80E-18	3.84E-18	3.84E-18	3.80E-18	3.51E-18	10/0
Vs. DE	2.42E-18	4.83E-19	1.52E-23	3.08E-18	1.53E-18	1.09E-18	2.48E-20	6.38E-20	5.30E-20	3.86E-18	10/0
Vs. GSA	3.61E-19	2.13E-19	3.61E-19	4.07E-19	3.61E-19	3.19E-19	5.10E-19	4.57E-19	3.19E-19	6.84E-20	10/0
Vs. MVO	3.90E-18	3.89E-18	3.89E-18	3.89E-18	3.90E-18	3.89E-18	3.90E-18	3.89E-18	3.89E-18	3.86E-18	10/0
Vs. PSO	3.41E-18	3.86E-18	3.78E-18	2.49E-18	3.81E-18	1.06E-18	3.13E-18	1.44E-22	2.63E-18	3.86E-18	10/0
Vs. SSA	3.64E-18	3.35E-18	3.61E-18	3.47E-18	3.35E-18	3.41E-18	3.71E-18	3.76E-18	3.23E-18	2.19E-18	10/0
Vs. GWO	3.90E-18	3.89E-18	3.90E-18	3.90E-18	3.90E-18	3.69E-05	3.90E-18	3.90E-18	3.90E-18	1.02E-05	10/0
Vs. EO	3.90E-18	3.89E-18	3.88E-18	3.89E-18	3.89E-18	8.27E-09	3.89E-18	3.87E-18	3.90E-18	8.95E-07	10/0
Vs. HHO	3.89E-18	3.86E-18	3.87E-18	3.67E-18	3.88E-18	3.88E-18	3.86E-18	3.83E-18	3.63E-18	3.85E-18	10/0
Vs. MPA	3.89E-18	3.61E-18	3.66E-18	3.87E-18	3.89E-18	1.48E-11	1.69E-04	3.07E-18	3.68E-18	3.84E-18	10/0
Vs. GJO	2.10E-19	1.49E-19	1.78E-18	**1.00E+00**	2.80E-18	**1.00E+00**	2.48E-20	**1.00E+00**	1.79E-18	3.34E-18	7/3

According to the statistical results in Table 14, when pairwise comparisons are made with other algorithms, the rank-sum test values of the GJO-GWO algorithm are significantly less than 0.05 for most datasets. This indicates that, in statistical terms, GJO-GWO demonstrates a significant advantage over the 13 comparison algorithms. This profound insight underscores the outstanding performance of GJO-GWO in feature selection problems, providing robust support for its reliability and effectiveness in practical applications.

### 5.3 Discussion

In Case 2, we conducted an in-depth investigation into the application of the GJO-GWO algorithm in combination with the KNN classifier for feature selection problems. We analyzed the experimental results on various complex datasets for 13 metaheuristic algorithms. Through this analysis, we gained a comprehensive understanding of the performance of the GJO-GWO algorithm when applied to feature selection problems. Here are the specific findings summarized below.

First and foremost, among the 13 metaheuristic algorithms applied to feature selection problems, the GJO-GWO algorithm demonstrates exceptional exploratory and exploitative performance. Its superior performance on high-dimensional datasets highlights its versatility in addressing FS problems. Additionally, the KNN-based GJO-GWO algorithm achieves higher classification accuracy and exhibits faster convergence on most datasets compared to other optimization algorithms. Lastly, the shorter average runtime implies that the KNN-based GJO-GWO algorithm is well-suited for swiftly solving complex feature selection problems.

While the KNN-based GJO-GWO algorithm generally produces efficient results for feature selection tasks, experiments reveal that it needs to excel in the number of features selected. These highlight feature selection algorithms’ inherent challenge in maintaining high classification accuracy while reducing the number of features. It emphasizes the existence of a trade-off between the number of features and classification accuracy, and selecting the most suitable feature selection method based on specific requirements can yield better results. Additionally, as the optimization results are inherently based on non-exact but repeatable processes, applying the GJO-GWO algorithm in various scenarios or problems may result in different feature subsets. Finally, it is essential to note that the KNN-based GJO-GWO feature selection algorithm, being a classical wrapper-based feature selection method, may exhibit variations in runtime, classification accuracy, and the number of selected features when used with different classifiers.

## 6. Conclusion and future directions

This research has been centered around addressing feature selection problems to improve the optimization performance of the GJO algorithm. Through mechanistic analysis and numerical experiments, the following conclusions have been drawn:

1. A multi-strategy integrated Golden Jackal-Gray Wolf Hybrid Optimization Algorithm (GJO-GWO) has been proposed. Compared with nine other metaheuristic algorithms on eight benchmark datasets, the proposed GJO-GWO exhibits significant advantages in terms of convergence and stability. These advantages mainly manifest in two aspects: a) Introducing the Gray Wolf Algorithm increases solution diversity. b) The position update strategy based on Lagrange interpolation enhances the algorithm’s convergence performance.
2. An efficient feature selection method based on GJO-GWO for classification tasks has been provided. On ten high-dimensional datasets, when compared to 13 state-of-the-art feature selection techniques, the proposed feature selection method demonstrates significant advantages in terms of accuracy, convergence speed, and runtime. These advantages mainly manifest in two aspects: a) Introducing the Gray Wolf Algorithm enhances solution diversity and improves the algorithm’s runtime efficiency due to its programming framework. b) The position update strategy based on Lagrange interpolation effectively increases the algorithm’s convergence speed. The clever integration of these strategies allows the algorithm to adaptively adjust the balance between exploration and exploitation at different search stages.

Despite the overall better performance of the feature selection method based on GJO-GWO presented in this paper, some things could still be improved. For instance, it often selects a relatively large number of features during feature selection for classification, which might not be ideal for subsequent machine learning or deep learning tasks. Therefore, we plan to conduct further research to address these issues in the future, as outlined below:

1. In the future, we intend to design a corresponding optimization and development framework around the GJO-GWO algorithm. This framework will be suitable for handling single or multi-objective optimization problems such as real-time feature selection, autonomous intelligent scheduling, image threshold segmentation, power system dispatch optimization, and neural network architecture search.
2. In the future, we aim to build a data mining and analytics system based on GJO-GWO. We will explore using the GJO-GWO algorithm or a combination of various metaheuristic algorithms as the underlying algorithms to create an integrated data mining and analytics system that encompasses feature engineering, parameter optimization, machine learning, deep learning, and decision optimization. This system will facilitate rapid analysis of real-world engineering application problems.

## Supporting information

S1 Data (ZIP)
